# BePresent Universal Internet-Based Parenting Intervention: Single-Arm Pre-Post Intervention Study

**DOI:** 10.2196/65391

**Published:** 2025-03-13

**Authors:** Kaisa Mishina, Amit Baumel, Malin Kinnunen, Terja Ristkari, Emmi Heinonen, Susanna Hinkka-Yli-Salomäki, Andre Sourander

**Affiliations:** 1 Research Centre for Child Psychiatry Faculty of Medicine University of Turku Turku Finland; 2 INVEST Research Flagship Centre University of Turku Turku Finland; 3 Department of Community Mental Health University of Haifa Haifa Israel; 4 Department of Child Psychiatry Turku University Hospital Turku Finland

**Keywords:** parent training, universal intervention, online intervention, irritability, conduct problems, hyperactivity, preschool, mental health, strongest families, positive parenting, parenting skills, parent-child relationships, parent satisfaction, BePresent, feasibility study, single-arm pre-post intervention study

## Abstract

**Background:**

Internet-based parenting programs have great potential to promote positive parent-child relationships as well as to reach and engage parents.

**Objective:**

This study aimed to assess the universal internet-based BePresent parenting intervention for families with 3-year-old children and how it influences the child’s behavior and daily-life situations assessed by parents. The first aim of the study was to assess the change from baseline to follow-up in child hyperactivity and conduct problems, affective reactivity, and daily activities. The second aim was to assess intervention completion rates. The third aim was to evaluate parent satisfaction with the intervention. The fourth aim was to assess all outcomes by comparing those who completed the intervention and those who did not.

**Methods:**

We conducted a single-arm pre- and postintervention study. Parents attending their child’s 3-year health check-up were recruited from children’s health clinics. The intervention was an unguided internet-based parenting program consisting of 5 modules. Self-reported measures were collected at baseline and at an 8-week follow-up. Linear mixed-effects models were used to analyze the changes from baseline to follow-up.

**Results:**

Altogether, 752 parents registered, and 515 started the intervention. Of those, 36% (n=183) completed the intervention. Parents reported high satisfaction with the intervention: the majority (68.8%–84.9%) were satisfied with various aspects of the program, and 89.9% said the intervention provided information about positive parenting skills. The findings show significant decreases with small effect sizes in parents’ ratings of child hyperactivity (*P*=.03; *d*=0.12) and conduct problems (*P*=.001; *d*=0.20) between baseline and the 8-week follow-up. A similar finding was observed in the parent ratings of child irritability (*P*≤.001; *d*=0.27) using the Affective Reactivity Index. Parents reported improvement in the daily functioning of their child when it was measured with a questionnaire adapted from the Barkley Home Situations Questionnaire (*P*=.01; *d*=0.14).

**Conclusions:**

Universal digital interventions have the potential to be implemented widely in community settings to improve knowledge and positive parenting skills. However, there is a need to assess the efficacy of digital universal interventions using randomized controlled designs and to examine additional ways to increase adherence to universal programs.

## Introduction

Consistent evidence has shown that parent training can lead to improvements in parenting practices and that it promotes the psychosocial development of children [1,2], which can, in turn, prevent various psychosocial and mental health difficulties [1,3]. Therefore, early preventive parent training programs are needed to enhance parent, and child relationships and to promote the healthy development of children [2].

Preventative interventions can be indicated, selective, or universal [4]. While indicated and selective interventions are intended for individuals with identified high risk, universal interventions can be offered to the general public or the entire population, typically regardless of individual risk factors [5]. Universal interventions aim to prevent the onset of illness or behavior by reducing exposure of risk factors [6]. In universal parent training, this means improving positive parenting [7], strengthening the relationships and interactions between parents and children [8], and reducing dysfunctional or abusive parenting practices [7]. In comparison to indicated or selective parenting programs, universal ones are typically briefer, targeting ordinary parenting challenges, and are hence aimed at all parents [9].

Universal programs typically have a large reach. They are considered to be less stigmatized, since any parent can experience parenting as challenging and thus need support [10]. Moreover, when universal parenting programs are offered in an internet-based format, typical barriers related to face-to-face programs can be avoided, such as logistical and financial barriers as well as the shortage of trained professionals [11]. Attrition rates in internet-based parenting programs typically vary between 30% and 50%, similar to that of face-to-face parenting programs [12], but internet-based programs generally have higher universal uptake [13].

A recent systematic review assessing universal digital parenting programs found small to moderate improvements in parent depression, anxiety, self-efficacy, and social support, but found no effects on parent’s stress, satisfaction, or parent-child relationship quality [11]. Due to limited data, the review was unable to assess the effects of universal digital parent training on child-specific outcomes [11], pointing out a clear research gap in the field. While the promise of universal parenting programs is conceptually clear, their impact on public health remains unclear.

This study focuses on the universal internet-based parenting program BePresent. The program is based on a wider 11-week targeted parent training program, Finnish Strongest Families, which has demonstrated efficacy in randomized controlled design up until a 2-year follow-up [14,15]. Since the randomized controlled study, the Strongest Families program has been implemented nationwide in Finland through child health clinics for parents whose 4-year-old children have disruptive behavior problems [16,17]. The parent training program has also been studied in clinical settings among 3 years-10 years–old children with disruptive behavior problems [18]. BePresent is a universal digital nonguided intervention aimed at all parents with 3-year-old children. Within the Finnish health system, 77% of preschoolers attend annual health check-ups at a child health clinic [19], which makes these visits an ideal setting for the delivery of such a universal intervention.

The first aim of the study was to assess changes in child hyperactivity and conduct problems, affective reactivity, and daily activities using a pre-post, single-arm intervention study design. The second aim was to assess intervention completion rates. The third aim was to evaluate parent satisfaction with the intervention. The fourth aim was to assess all outcomes by comparing those who completed the intervention and those who did not.

## Methods

### Study Design

A single-arm pre- and postintervention study involved the recruitment of participants to use the BePresent parenting intervention. Measures were conducted at baseline before starting the intervention and at an 8-week follow-up after filling in the baseline questionnaire.

### Intervention

BePresent is a universal, unguided internet-based parenting intervention. The content and structure of the intervention are based on the Finnish Strongest Families targeted parent training program. Patterson’s Coercion Theory guided the development, emphasizing the importance of positive reinforcement in parent-child interactions. The universal intervention follows the basics of the targeted program but was shortened to be feasible for all parents with 3-year-old children. BePresent focuses on the promotion of healthy parenting practices and developing parenting skills that strengthen the relationship and communication with the child. The topics include reinforcing the child’s positive behavior, reducing conflict situations, planning situations in advance, managing daily transitions, and being emotionally present with the child. The intervention includes 5 modules, and the total intervention period is 8 weeks. Detailed information about the key training elements and parental goals of each module are presented in Table 1.

**Figure 1:**
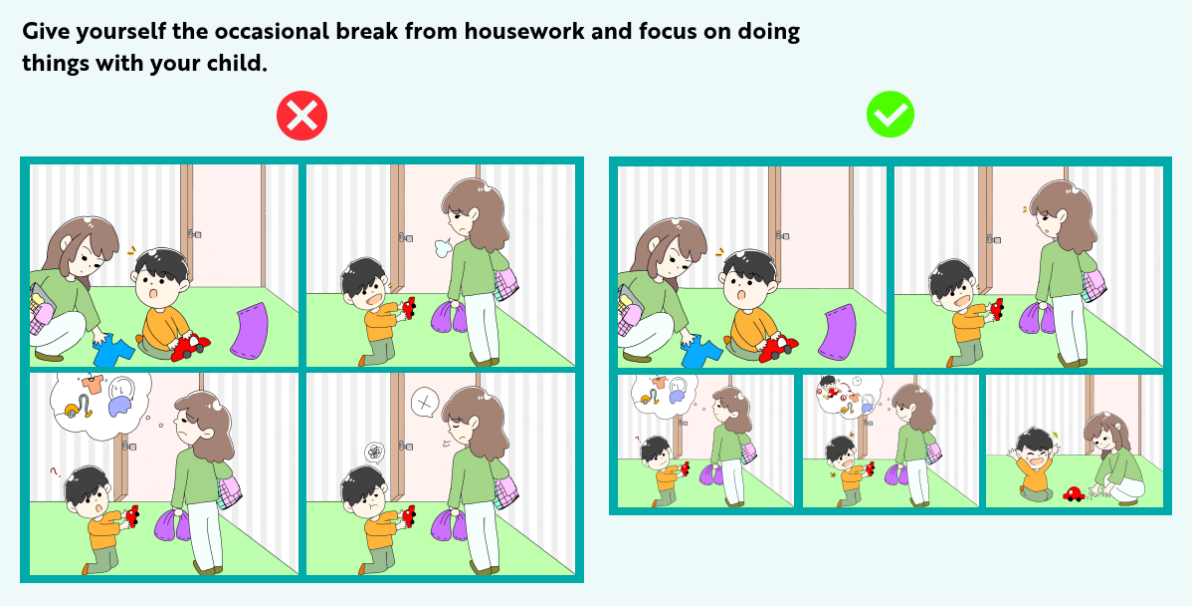
Negative and positive examples of how to be more present with your child in daily situations.

The participants must complete the modules sequentially; they cannot select which module they start with. Each module includes theory material, example videos, comic strips (Figure 1), video practices, and exercises. In the comic strips, the central ideas of the program’s example videos are presented. For example, in module 5 the importance of spending time with the child, for example, by playing, is presented in the video and in the comic strip. In both, first, a negative situation is presented, followed by a positive example of parents using the practiced parenting skills. The comic strips use visual-driven storytelling, including pictures without text. With this, we wanted to convey a universal understanding, across different languages. There are different variations of individual strategies to choose from to train parenting skills in practice. To motivate and promote adherence to the program, standardized and same-for-all SMS and email reminders were sent regularly during the program. The participants had 8 weeks to complete the intervention. After this, they were asked to fill out the follow-up questionnaire.

**Table 1:** Structure and content of the BePresent internet-based parenting program.

Module	Key training elements	Parental goals
Notice the good in your child	Positive attention Positive feedback Reducing unnecessary reminders	Positive interaction skills with child Active parenting
Understand your child	Acting in daily situations Boundaries for the child Self-controlled parenting	Self-regulation with child Staying calm Recognizing negative thoughts Turning thoughts into more positive
Prepare your child for changes	Modeling daily transitions Importance of daily routines	Reinforcing good daily routines
Plan ahead with your child	Planning together with the child Preparing a child for upcoming situations	Involving the child in planning and reinforce good daily routines
Be present for your child	Conscious presence with the child Spending time with the child Mindfulness	Being present in the daily moments with the child

### Participants and Setting

Parents with a 3-year-old child participating in annual health check-ups in primary care child health clinics in Finland were considered eligible and were asked to participate. The program was offered in Finnish, Swedish, and English; therefore, at least one of the parents had to understand one of those languages for the family to participate. The study setting was the public child health clinics, and the health check-ups offered there are free of charge for the families. Out of the 21 well-being services counties in Finland, 14 (67%) were included in this study. These well-being service counties were included as they were a part of our broader implementation study, selected to represent larger and smaller cities in both urban and rural areas across Finland. Since the operations of child health clinics are regulated by the Finnish Health Care Act [20] and the Government Decree [21], quality and operations are relatively uniform across the counties.

### Procedure

There were 2 paths to participating in the BePresent program. First, as a part of the annual health check-up, a public health nurse informed parents about the program and the study. Information was also provided in written format on a flyer that the public health nurse gave to the parents during the check-up. The flyer included the program’s web address, where the parents could sign in and complete the registration form (name, email, and phone number). Second, 3 well-being services counties also recruited parents to the BePresent intervention through their website and social media pages. This internet-based information was similar to that on the flyer. A link was provided that, when followed, led parents to a form that screened them for inclusion criteria through a series of questions (which well-being services county did they live in; were they a guardian of a 3-year-old; did they understand Finnish, Swedish, and English). If the parents fulfilled the inclusion criteria, registration instructions were automatically sent to them by email.

The parents were asked to register for the intervention 1 month after the health check-up at the latest, but the registration stayed open until the end of the study. The website included information about the study and the intervention as well as an electronic informed consent form. After giving informed consent, parents were asked to fill in a baseline evaluation. The intervention lasted for 8 weeks. After this, parents were asked to complete a follow-up questionnaire; they also received email and text message reminders about the follow-up questionnaire. Intervention completers were defined as parents who completed the first 4 modules since those modules included the key elements of the parent training. The fifth theme concentrated on conscious presence with the child; this was considered to be an additional skill.

### Measures

#### Demographic Background Information

To gather demographic background information, participating parents were asked about their gender, their child’s sex, their family structure, and their role in the child’s life. The question about family structure referred to who lived with the child, and the response options were (1) both biological parents, (2) the biological mother or father and their spouse, (3) the biological mother or father alone, (4) adoptive parents, (5) foster parents, (6) parents with the same gender, or (7) some other type of family. In the analysis, family types other than those with both biological parents were pooled together.

#### Child Hyperactivity and Conduct Problems

Hyperactivity and conduct problems were assessed with scales based on the content of subscales of the Strengths and Difficulties Questionnaire [22,23]. The Strengths and Difficulties Questionnaire internalizing subscales were not included in the questionnaire because the aim was to have as brief of a questionnaire as possible. Altogether, the scales measuring conduct problems and hyperactivity consisted of 10 items. Responses were based on the format 0=not true, 1=somewhat true, and 2=certainly true, so the scores for each scale of 5 items could range from 0 to 10. In the current study, the Cronbach alpha was 0.77 and the McDonald’s omega was 0.79 for hyperactivity and .69 (α) and 0.70 (ω) for conduct problems, indicating acceptable reliability [24,25].

#### Child Affective Reactivity

The affective reactivity index-P [26] is a 7-item instrument measuring the irritability of a child. The affective reactivity index-P has been proven to be valid for measuring parent-rated irritability among small children [27]. Each item could be responded to using a scale of 0 to 2, indicating the responses of not true, somewhat true, or certainly true, respectively. The total score was calculated as the sum of the first 6 items, meaning that the total score range was 0-12. The impairment item was not counted in the total score. The McDonald’s omega was 0.86 and the Cronbach α was 0.84, indicating acceptable reliability [24,25].

#### Daily Activities

Daily activities were assessed by asking the parents to rate the impact of the child’s behavior during daily transitions, including getting dressed, getting ready for daycare, during the evening meal, and getting ready for bed. It also covered social interactions, including playing with siblings and other children, traveling situations, and being in public places such as the supermarket. The questionnaire was adapted from the Barkley Home Situations Questionnaire, which asks the parent to rate whether the child’s behavior causes problems during specified daily routines [28]. The instrument has been previously used in the Finnish Strongest Families parent training program when it presented adequate reliability with a Cronbach α of 0.64 [17]. In the current study, the McDonald’s omega was 0.87 and the Cronbach α was 0.87, indicating acceptable reliability [24,25].

### Intervention Completion

Completion and non-completion of the intervention for the 8-week period was assessed using the data recorded by the intervention platform. Intervention completers were defined as parents who completed module 4, as the first 4 modules included the key elements of the intervention. Participation in the follow-up measures was also assessed using the data from the intervention platform.

### Intervention Satisfaction

Participants were asked to rate their satisfaction with the intervention with 1 item, with the response options being 0=very dissatisfied, 1=somewhat dissatisfied, 2=neither dissatisfied nor satisfied, 3=somewhat satisfied, and 4=very satisfied. In the analysis, the options were categorized as dissatisfied, neither dissatisfied nor satisfied, and satisfied. The questionnaire included 10 items used to rate how well participants felt the intervention provided support in specific areas of parenting. The response options were 0=disagree, 1=somewhat disagree, 2=neither disagree nor agree, 3=somewhat agree, and 4=agree. In the analysis, the responses were categorized as disagree, neither disagree nor agree, and agree.

### Statistical Analysis

Demographic characteristics and satisfaction with the program are presented as numbers and percentages. The difference between groups of completers and noncompleters were tested with Pearson’s chi-squared test. The changes in outcome variables from baseline to the 8-week follow-up (with the unit of the analyses being parents’ ratings of their children) were analyzed with linear mixed effect models for those who completed the follow-up questionnaire, with time as the within factor (baseline and 8-week follow-up) and the child’s sex as a covariate. The differences in outcome variables between completers and noncompleters at the 8-week follow-up were analyzed with linear regression models. Because the baseline values differed between groups, the models were adjusted for the baseline measurement of the outcome and the child’s sex. A 2-sided significance level of .05 was used during the statistical testing, and 95% Cl were calculated for the point estimates. Cohen *d* was calculated as a measure of effect size, to complement the statistical testing. The statistical analyses were carried out with SAS statistical software (SAS 9.4, SAS Institute).

### Ethical Considerations

All study procedures and human subject research ethics were approved by the Ethical Committee of the University of Turku (45/2017, 11 Sep 2017). Study permission was granted from each study site, including Wellbeing Services County of South Karelia, Wellbeing Services County of South Ostrobothnia, South Savo Wellbeing Services County, Wellbeing Services County of Kymenlaakso, Wellbeing Services County of Lapland, Western Uusimaa Wellbeing Services County, Wellbeing Services County of Pirkanmaa, Wellbeing Services County of Ostrobothnia, Wellbeing Services County of Satakunta, Wellbeing Services County of North Karelia, Wellbeing Services County of Central Ostrobothnia, Wellbeing Services County of Vantaa and Kerava. and Wellbeing Services County of Southwest Finland. The participants provided informed consent before enrollment in the study. No form of compensation, monetary or otherwise, was offered to participants. Privacy and confidentiality of the participants was ensured by adhering to ethical guidelines, securely handling data and pseudonymizing identifiable information. The data was pseudonymized and stored in the University of Turku secured project folder (short term) with automatic backup system and in “Taltio” and/or “Isilon” data storage cluster (long term). We followed the TREND (Transparent Reporting of Evaluations with Nonrandomized Designs) Statement Checklist for complete and detailed reporting (Multimedia Appendix 1).

## Results

**Figure 2:**
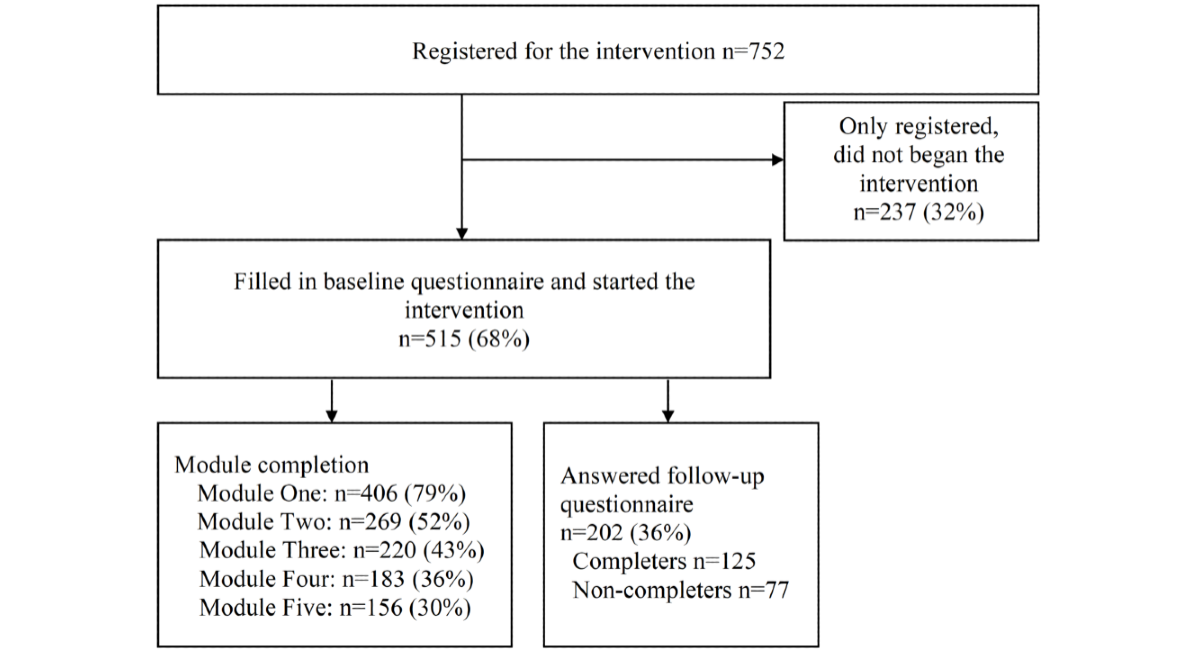
Flow chart of the study participants and information on completers and noncompleters. Began Module 1 n=515. Completed first 4 modules n=125. Started the intervention but did not complete the first 4 modules n=77.

Detailed information about enrolment, participation per module, and completion rates of the modules are presented in Figure 2. Altogether, 752 parents registered for the BePresent parenting intervention and 515 started the intervention; 32% registered but did not begin the intervention. Out of the 515 who started the intervention, 202 (36%) answered the follow-up questionnaire, and 183 (36%) completed the intervention. The highest dropout rate occurred between the first and second modules, with a dropout rate of approximately 34%.

Table 2 presents the participants’ background characteristics and a comparison between those of completers and noncompleters. The majority of participating parents were mothers (90.9%) and most of the children were boys (57.9%). Most families had a structure including both biological parents (92.2%). The percentage of completers who were boys was significantly higher than that of noncompleters who were boys (67.8% vs 52.4%, *P*<.001). No other demographic difference was found between intervention completers and noncompleters. The average time spent on the intervention website was 8.3 hours among those who completed the intervention and 3.5 hours among noncompleters (Table 3).

**Table 2:** Background characteristics and comparison between completers and noncompleters.

Characteristics		Total (N=515), n (%)			Completers (n=183), n (%)			Noncompleters (n=332), n (%)		
**Child’s sex**										
	Boy		298 (57.9)			124 (67.8)			174 (52.4)	
	Girl		217 (42.1)			59 (32.2)			158 (47.6)	
**Family structure**										
	Both biological parents			475 (92.2)			170 (92.9)			305 (91.9)
	Other			40 (7.8)			13 (7.1)			27 (8.1)
**Participant’s gender^a^**										
	Women		463 (89.9)			164 (89.6)			299 (90.1)	
	Men		46 (8.9)			16 (8.7)			30 (9.0)	
	Not specified		6 (1.2)			3 (1.6)			3 (0.9)	

^a^Gender of the parent who registered for the program and responded to the questionnaires.

**Table 3:** Intervention usage of the participants and comparison between completers and noncompleters.

Characteristics			Total (N=515)				Completers (n=183)		Noncompleters (n=332)				Completers versus noncompleters (*P* value^a^)
**Intervention usage, mean (SD)**													
	Number of days completing the intervention (days)			19.1 (22.3)	28.6 (19.6)					11.2 (21.3)	<.001		
	Number of days per completed module (days)			6.4 (12.0)	5.9 (4.1)					6.8 (15.7)	.45		
	Active use of website per completed module (hours)			2.1 (1.6)	1.7 (1.2)					2.4 (1.8)	<.001		
	Active total use of the website (hours)			5.7 (4.9)	8.3 (5.7)					3.5 (2.8)	<.001		
**Time from beginning the module to completing the module (hours, median)**													
	Module 1			0.24	—^b^					—	—		
	Module 2			0.19	—					—	—		
	Module 3			0.11	—					—	—		
	Module 4	0.16				—		—				—	

^a^Student *t* test.

^b^Not applicable.

### Behavioral Outcomes

Table 4 presents a sample including completers and noncompleters, showing the changes in child behavior and daily situations from baseline to the follow-up measurement for those who completed the measures at both time points (n=202). There were significant improvements from baseline to the 8-week follow-up in hyperactivity (*P*=.03; *d*=0.11) and conduct problems (*P*=.001; *d*=0.20). Child affective reactivity also improved significantly (*P*<.001; *d*=0.26) from baseline to follow-up. In daily situations, improvements were found in total scores (*P*=.01; *d*=0.14) as well as in transition (*P*<.001; *d*=0.25) and eating situations (*P*=.002; *d*=0.19).

While Table 4 includes results for completers and noncompleters combined, Table 5 presents a comparison between the 2 groups and a more detailed description of changes among completers and among noncompleters from baseline to follow-up. As presented in Table 5, affective reactivity improved more among noncompleters than among completers (*P*=.03; *d*=0.21). At baseline, completers had more difficulties in affective reactivity, hyperactivity, and conduct problems than noncompleters.

**Table 4:** Comparing hyperactivity and conduct problems, affective reactivity, and daily situations mean scores between baseline and the 8-week follow-up among those who completed the baseline and the follow-up questionnaire, adjusted by the children’s gender.

Total (n=202)^a^				Baseline, mean^b^ (SE)			Follow-up, mean^b^ (SE)		Change from baseline to 8-week follow-up				
									Mean change value^b^ (95% CI)		*P* value		Cohen *d* (95% CI)
**Behavioral outcome (n=198)**													
	Hyperactivity^a,d^		3.9 (0.2)			3.7 (0.2)		0.3 (0.0 to 0.5)		.03		0.11 (–0.08 to 0.31)	
		Conduct problems^a,d^	3.6 (0.1)			3.2 (0.1)		0.4 (0.2 to 0.6)		.001		0.20 (–0.001 to 0.40)	
**Affective Reactivity (n=202)**													
	Total		4.2 (0.2)		3.4 (0.2)			0.8 (0.5 to 1.1)		<.001		0.26 (0.06 to 0.46)	
**Daily situations (n=190)**													
	Total score		35.3 (0.7)			34.0 (0.7)		1.3 (0.3 to 2.3)		.01		0.14 (–0.06 to 0.33)	
	Transition situations		12.7 (0.3)		11.7 (0.3)			1.0 (0.5 to 1.5)		<.001		0.25 (0.05 to 0.45)	
	Eating situations		7.2 (0.2)		6.6 (0.2)			0.5 (0.2 to 0.8)		.002		0.19 (–0.01 to 0.39)	
	Situations outside home		7.5 (0.2)		7.6 (0.2)			–0.1 (–0.4 to 0.2)		.67		–0.03 (–0.23 to 0.18)	
	Situations inside home		7.9 (0.2)		8.1 (0.2)			–0.2 (–0.5 to 0.2)		.42		–0.05 (–0.25 to 0.15)	

^a^Includes both intervention completers and noncompleters.

^b^Model based on least squares means.

**Table 5:** Comparing baseline and follow-up changes in hyperactivity, conduct problems, affective reactivity, and daily situations between intervention completers and noncompleters.

				Completers (N=125)									Noncompleters (n=77)									Completers versus noncompleters				
				Baseline, mean^a^ (SE)		Follow-up, mean^a^ (SE)		Change^b^				Baseline, mean^a^ (SE)			Follow-up, mean^a^ (SE)		Change^b^									
								Mean^a^ (95% CI)		*P* value							Mean^a^ (95% CI)		*P* value		Mean^b,c^ (95% CI)			*P* value		Cohen *d* (95% CI)
**Behavioral outcome (n=198)**																										
		Hyperactivity	4.1 (0.2)		3.8 (0.2)		0.3 (0.0 to 0.7)		.04		3.6 (0.3)			3.4 (0.3)		0.2 (–0.3 to 0.6)		.44		0.0 (–0.5 to 0.5)			.96		0.00 (–0.19 to 0.20)	
		Conduct problems	3.7 (0.2)		3.3 (0.2)		0.4 (0.1 to 0.7)		.006		3.4 (0.2)			3.0 (0.2)		0.4 (–0.0 to 0.7)		.077		0.0 (–0.5 to 0.5)			.98		0.00 (–0.19 to 0.20)	
**Affective reactivity (n=202)**																										
	Total		4.4 (0.3)		3.7 (0.3)		0.6 (0.2 to 1.1)		.006		4.0 (0.3)			3.0 (0.2)		1.0 (0.6 to 1.4)		<.001		0.6 (0.0 to 1.1)			.03		0.21 (0.02 to 0.41)	
**Daily situations (n=190)**																										
	Total score		35.6 (0.9)		34.0 (0.9)		1.5 (0.3 to 2.8)		.0 13		34.5 (1.0)			33.7 (1.0)		0.8 (–1.1 to 2.7)		.39		–0.2 (–2.2 to 1.9)			.8 8		–0.01 (–0.22 to 0.19	
	Transition situations		13.1 (0.4)		12.0 (0.4)		1.1 (0.5 to 1.7)		<.001		11.9 (0.4)			11.1 (0.4)		0.8 (–0.0 to 1.6)		.06		0.2 (– 0.7 to 1.1)			.6 3		0.05 (–0.15 to 0.25)	
	Eating situations		7.1 (0.3)		6.7 (0.3)		0.4 (0.0 to 0.8)		.03		7.1 (0.3)			6.5 (0.3)		0.7 (0.1 to 1.2)		.02		0.3 (–0.3 to 0.9)			.26		0.11 (–0.09 to 0.32)	
	Situations outside home		7.4 (0.2)		7.4 (0.2)		0.0 (–0.4 to 0.4)		1.00 0		7.6 (0.3)			7.8 (0.3)		–0.2 (–0.8 to 0.4)		.510		–0.2 (–0.8 to 0.4)			.54		–0.06 (–0.26 to 0.14)	
	Situations inside home		8.0 (0.3)		8.0 (0.3)		0.0 (–0.4 to 0.4)		.97		7.9 (0.3)			8.3 (0.4)		–0.5 (–1.2 to 0.3)		.21		–0.3 (–1.0 to 0.4)			.42		–0.08 (–0.28 to 0.12)	

^a^Model based least squares means.

^b^Change from baseline to follow-up.

^c^Adjusted with corresponding baseline and child’s sex.

### Intervention Satisfaction

Parents reported high levels of satisfaction with the program (Table 6). Over 80% of the parents indicated that they would recommend the program to others and agreed that the program provided information about positive parenting skills as well as ways to notice the good in their child. Satisfaction was significantly higher among those who completed the program.

**Table 6:** Program satisfaction and acknowledged support in specific areas of parenting and comparison between completers and noncompleters.

Satisfaction-related factors^a^				Total (N=186), n (%)					Completers (n=123), n (%)					Noncompleters (n=63), n (%)					Completers versus noncompleters (*P* value)		
**Satisfaction**																					
	**The program met my expectations**																			.001	
		Agree				142 (76.3)					104 (84.6)					38 (60.3)					
		Neutral				27 (14.5)					13 (10.0)					16 (25.4)					
		Disagree				17 (9.1)					11 (8.9)					9 (14.3)					
	**The program suited my needs**																			.005	
		Agree			141 (75.8)					102 (82.9)					39 (61.9)						
		Neutral	21 (11.3)					11 (8.9)					10 (15.9)								
		Disagree	24 (12.9)					10 (8.1)					14 (22.2)								
	**I could recommend the program to others**																			<.001	
		Agree	158 (84.9)					114 (92.7)					44 (69.8)								
		Neutral	20 (10.8)					6 (4.9)					14 (22.2)								
		Disagree	8 (4.3)					3 (2.4)					5 (7.9)								
	**I could join the program again**																			.10	
		Agree	147 (79.0)					102 (82.9)					45 (71.4)								
		Neutral	21 (11.3)					13 (10.6)					8 (12.7)								
		Disagree	18 (9.7)					8 (6.5)					10 (15.9)								
	**How satisfied you have been with the program**																			.003	
		Satisfied				128 (68.8)					94 (76.4)					34 (54.0)					
		Neutral				37 (19.9)					21 (17.1)					13 (23.2)					
		Dissatisfied				21 (11.3)					8 (6.5)					16 (25.4)					
**Support in specific areas**																					
	**The program provided information about positive parenting skills**																			<.001	
		Agree				167 (89.8)					119 (96.7)					48 (76.2)					
		Neutral				12 (6.5)					1 (0.8)					11 (17.5)					
		Disagree				7 (3.8)					3 (2.4)					4 (6.3)					
	**The program offered ways to notice the good in my child**																			<.001	
		Agree					164 (88.2)					116 (94.3)					48 (76.2)				
		Neutral					13 (7.0)					3 (2.4)					10 (15.9)				
		Disagree					9 (4.8)					4 (3.3)					5 (7.9)				
	**The program gave me confidence in my ability to be a parent**																			.002	
		Agree	146 (78.5)					106 (86.2)					40 (63.5)								
		Neutral	27 (14.5)					12 (9.8)					15 (23.8)								
		Disagree	13 (7.0)					5 (4.1)					8 (12.7)								
	**The program offered ways to manage everyday situations**																			<.001	
		Agree	144 (77.4)					106 (86.2)					38 (60.3)								
		Neutral	26 (14.0)					12 (9.2)					16 (25.4)								
		Disagree	16 (8.6)					10 (8.1)					9 (14.3)								
	**The program offered ways to manage transitions**																			<.001	
		Agree	134 (72.0)					99 (80.5)					35 (55.6)								
		Neutral	34 (18.3)					14 (11.4)					20 (31.7)								
		Disagree	18 (9.7)					10 (8.1)					8 (12.7)								
	**The program offered ways to be present with my child**																			.002	
		Agree			146 (78.5)					106 (86.2)					40 (63.5)						
		Neutral	32 (17.2)					16 (12.3)					19 (30.2)								
		Disagree	8 (4.3)					4 (3.3)					4 (6.3)								

^a^Disagree combines strongly disagree and disagree. Agree combines agree and strongly agree.

## Discussion

### Principal Findings

In this study, we assessed a universal unguided internet-based parenting intervention and whether it improves child behavior and daily activities. Hyperactivity, conduct problems, affective reactivity, and daily activities improved from baseline to follow-up, and parents reported a high level of satisfaction with the intervention and showed rather good adherence to it. Out of all the parents who started using the intervention, 36% (n=183) completed it.

The outcomes were also assessed by comparing those who completed the intervention and those who did not complete it, and we found that child irritability improved among the completers and the noncompleters. This finding is important; interventions targeting small children’s irritability are central, as irritability in childhood predicts mental health problems, functional impairment, and outpatient treatment use in later life [29]. However, at the moment the number of studies on the topic is limited, especially studies on parent training for decreasing irritability [30]. Interestingly, noncompleters demonstrated greater improvement in affective reactivity compared with completers, and, at baseline, completers had more difficulties in affective reactivity. This may suggest that parents of children with greater initial difficulties were more motivated to engage with the program. It is also possible that this counterintuitive finding was because more severe difficulties are inherently harder to improve than milder ones. In this study, we also found decreases in child hyperactivity and conduct problems with small effect sizes.

Although effect sizes in universal programs are typically smaller than in targeted programs [31], our findings are encouraging and may reflect that universal internet-based interventions can improve child-specific mental health outcomes. In addition, internet-based parenting programs are effective for several parenting outcomes. A meta-analysis of parenting programs by Spencer et al [32] found no significant differences when comparing programs that included clinical support to programs that only contained internet-based components. Moreover, the parents reported high satisfaction with the interventions and felt that the interventions gave them confidence in their parenting capabilities, which is in line with previous studies in this field. Thus, considering the limited resources in society, internet-based parenting interventions may offer a wise way of supporting parenting.

Of the parents who started using the intervention, 36% completed it, forming an attrition rate of 64%. Previous studies on universal unguided parenting programs have reported similar attrition rates. For example, in the Australian internet-based self-directed parenting program ParentWorks, which included six sequenced modules, Dadds et al [13] found 68% attrition up until module 4 of the program. They defined full completion of the intervention as completing at least 5 core modules and the post-intervention survey. Among their 2967 parent participants, 8% were classified as full completers. In contrast, 17% of the participants in our study fully completed the intervention, resulting in a higher proportion of parents completing the entire program. One possible explanation for this discrepancy is that Dadds et al [13] only invited participants who had completed the core modules to fill out the postintervention survey, whereas in our study, the follow-up questionnaire was sent to all participants regardless of their completion status. ParentWorks was free of charge, and parents were recruited through a national media campaign and flyers distributed to child and family services. In another example, from the United States, which recruited parents from a well-child visit in primary care, Breitenstein et al [33] assessed a digitally delivered, self-administered parent training intervention, ezParent, and found that, out of the program’s 6 modules, 33% of parents completed at least 4 modules. The completion rate is comparable to our study, in which 36% of parents who began the BePresent program completed at least 4 modules. This suggests that completion rates below 40% may be relatively typical for web-based modules, unguided universal parenting programs.

The main reasons for discontinuing the use of a universal parenting program include the parent being too busy or finding the program unhelpful [34]. In fact, a typical disadvantage of universal programs is their tendency to lose sufficient focus on the individualized needs of users, causing a modest matching of program goals with individual needs [35]. In our universal intervention, simple personalized and customized strategies were used, such as the possibility to choose individual strategies to complete the exercises. However, the main parts of the intervention were the same for everyone. Parenting programs that address families’ actual needs facilitate program adherence [36], and personalized approaches could extend the program completion for those who currently do not benefit from such programs [37]. For example, in a recent pilot trial, Baumel et al [38] found that the program completion of an unguided digital parent training program aimed at treating child disruptive behaviors increased from 28% in a standard version (12/43 of participating parents) to 69% (31/45 of participating parents) in an enhanced version, through the use of features such as intervention tailoring, timely reminders, automated monitoring, and feedback. BePresent had sequential modules, meaning that the modules were in a defined order and the order was the same for everyone.

Based on the findings of this study, some potential strategies to improve retention in universal internet-based parenting programs can be identified. First, motivation could potentially be increased and attrition rates decreased if programs allowed parents to choose the modules they felt would best benefit their situation and personalize the modules according to their needs. Second, tailored support, such as frequent and personalized reminders could motivate parents to use the program. These reminders could be based on parents’ progress in the program or their specific needs. Third, it seems that the most common point for discontinuing the program was between modules 1 and 2, with a dropout rate of approximately 34%. Engagement should be especially supported here. Encouraging actions, especially at the beginning of the program might be highly beneficial. Universal parenting programs could benefit from evolving technology to provide more personalized approaches. For example, certain modules and different program lengths could be suggested based on parents’ reports of their needs and the skills they already possess.

The current study and previous studies about universal parenting programs have shown short-term improvements in individual-level outcomes, but it is important to notice that universal interventions can also foster changes in public health at the population level, too. This means focusing on prevention at the population level, for example, by increasing knowledge or changing attitudes [39]. From a parent training perspective, universal programs can aim to prevent problems in parenting and in child behavior, and the results can be seen in the number of those who eventually seek treatment. However, when aiming for population-level changes, the reach of the intervention must be comprehensive enough. Universal interventions typically have low penetration rates, mainly because it is a family’s own responsibility to seek out the intervention [35]. Our parent training program was offered when families attended a free of charge annual health check-up, something that all families in Finland are invited to participate in. Using this kind of setting, participation in the intervention was designed to be as easy as possible. Reach and easiness to participation should be carefully considered when implementing universal parenting programs. In addition, it would be important to also assess the population-level effects of universal parenting programs rather than focusing only on individual-level outcomes [39].

### Limitations

The study has several limitations to consider. First, this was not a randomized controlled study; therefore, effectiveness and efficacy could not be assessed. Second, it was not possible for us to assess how many parents were offered the chance to participate in the program. The child health clinics were instructed to offer the program to all parents who fulfilled the inclusion criteria. Third, because this was an acceptability study, we were not aiming to reach the population level, so the reach of this study was limited. However, the program was studied in a wide geographical area, covering 67% of well-being services counties in Finland, making the study geographically highly representative. Fourth, the effect sizes seen in this study are low, reaching a Cohen *d* of 0.27, at the highest. The effect sizes in previous universal internet-based parenting interventions have been small to moderate [11]. Effect sizes of parent training trials based on clinical samples have typically been higher, including large effects [40]. One reason for the low effect size in our study was that the level of problems among the sample, taken from the general population, was less severe than that of clinical samples, indicating that there was less room for improvement. Fifth, the study did not ask participants their reasons for not completing the intervention; therefore, this could not be factored or taken into account in our analysis.

### Conclusions

The universal internet-based parenting program BePresent may promote improvements in behavior and the daily lives of children. The completion rate was similar to that of other internet-based unguided universal parenting programs. In addition, parents were satisfied with the program and perceived that it supported their positive parenting skills. This type of program is low in cost, requires minimal resources, and can produce several benefits. Therefore, programs like this have the potentiality to be implemented widely in community settings to improve knowledge and positive parenting skills. However, there is a need to assess the impact of personalizing digital universal interventions to increase program adherence and to assess the efficacy of such programs in randomized controlled designs.

## Appendix

Multimedia Appendix 1 TREND (Transparent Reporting of Evaluations with Nonrandomized Designs) Statement Checklist for complete and detailed reporting.

## Abbreviations

TREND: Transparent Reporting of Evaluations with Nonrandomized Designs

## Data Availability

The datasets generated or analyzed during this study are available from the corresponding authors on reasonable requests.
